# Intra-individual comparison of ^68^Ga-PSMA-11-PET/CT and multi-parametric MR for imaging of primary prostate cancer

**DOI:** 10.1007/s00259-016-3346-0

**Published:** 2016-03-14

**Authors:** F. L. Giesel, F. Sterzing, H. P. Schlemmer, T. Holland-Letz, W. Mier, M. Rius, A. Afshar-Oromieh, K. Kopka, J. Debus, U. Haberkorn, C. Kratochwil

**Affiliations:** Department of Nuclear Medicine, University Hospital Heidelberg, INF 400, 69120 Heidelberg, Germany; Department of RadioOncology, University Hospital Heidelberg, Heidelberg, Germany; Department of Radiology, German Cancer Research Center (DKFZ), Heidelberg, Germany; Department of Biostatistics, German Cancer Research Center (DKFZ), Heidelberg, Germany; Institute of Transuranium Elements, European Commission (EC), Karlsruhe, Germany; Unit of Radiopharmaceutic Chemistry, German Cancer Research Center, Heidelberg, Germany; Division of Radiopharmaceutical Chemistry, German Cancer Research Center (DKFZ), Heidelberg, Germany

**Keywords:** Prostate cancer, PSMA, Multi-parametric MRI, T-staging

## Abstract

**Purpose:**

Multi-parametric magnetic resonance imaging (MP-MRI) is currently the most comprehensive work up for non-invasive primary tumor staging of prostate cancer (PCa). Prostate-specific membrane antigen (PSMA)-Positron emission tomography–computed tomography (PET/CT) is presented to be a highly promising new technique for N- and M-staging in recurrent PCa-patients. The actual investigation analyses the potential of ^68^Ga-PSMA11-PET/CT to assess the extent of primary prostate cancer by intra-individual comparison to MP-MRI.

**Methods:**

In a retrospective study, ten patients with primary PCa underwent MP-MRI and PSMA-PET/CT for initial staging. All tumors were proven histopathological by biopsy. Image analysis was done in a quantitative (SUVmax) and qualitative (blinded read) fashion based on PI-RADS. The PI-RADS schema was then translated into a 3D-matrix and the euclidian distance of this coordinate system was used to quantify the extend of agreement.

**Results:**

Both MP-MRI and PSMA-PET/CT presented a good allocation of the PCa, which was also in concordance to the tumor location validated in eight-segment resolution by biopsy. An Isocontour of 50 % SUVmax in PSMA-PET resulted in visually concordant tumor extension in comparison to MP-MRI (T2w and DWI). For 89.4 % of sections containing a tumor according to MP-MRI, the tumor was also identified in total or near-total agreement (euclidian distance ≤1) by PSMA-PET. Vice versa for 96.8 % of the sections identified as tumor bearing by PSMA-PET the tumor was also found in total or near-total agreement by MP-MRI.

**Conclusions:**

PSMA-PET/CT and MP-MRI correlated well with regard to tumor allocation in patients with a high pre-test probability for large tumors. Further research will be needed to evaluate its value in challenging situation such as prostatitis or after repeated negative biopsies.

## Introduction

Initial prostate cancer staging is an important diagnostic procedure for patient stratification. During the last years, non-invasive multi-parametric magnetic resonance imaging has been introduced and clinically well established for T-staging in prostate cancer patients [1]. Multi-parametric magnetic resonance imaging (MP-MRI) includes several different MR-sequences, i.e., T2w, DW, DCE-MRI and MRS. Due to a very high imaging data volume, reporting standards such as PI-RADS were introduced to harmonize image analysis and improve patient staging outcome [2].

Recently, prostate-specific membrane antigen (PSMA)-Positron emission tomography–computed tomography (PET/CT) based on the ligand ^68^Ga-PSMA11 has shown the potential to improve the detection of metastatic spread of prostate cancer in patients with biochemical recurrence [3, 4]. In this setting, PSMA-PET/CT was found superior in comparison to contrast-enhanced CT [5, 6] and Choline-PET/CT [7] with regard to lymph node involvement and detection of distant metastases. Phase-I/II data found PSMA-SPECT/CT with ^99m^Tc-trofolastat (MIP-1404) is also promising for diagnostics of primary PCa [8]. In contrast, data for T-staging with PSMA-PET/CT have not been reported as of yet. Thus, MRI is still considered as the gold standard for T-Staging of PCa, due to the fact of detailed depiction of size, infiltration and adjacent organ involvement [1, 2].

This retrospective study aimed to quantify the agreement of multi-parametric MRI and PSMA-PET/CT in T-Staging.

## Material and methods

### Patients

This is a retrospective analysis of ten patients scheduled for primary radiotherapy of their PCa who received both multiparametric magnetic imaging and PSMA-PET/CT imaging. External beam radiotherapy of localized prostate cancer is not inferior to radical prostatectomy [9]. For this collective of patients, imaging of primary PCa is of high clinical impact for defining the target volume of radiotherapy. In contrast, surgery candidates rarely benefit from improved local imaging; thus, we were not able to include surgery patients who would allow a better correlation to histopathology.

The median age was 70 years (range 61–74) with a median Gleason score (GSC) of 8 (range 7b–9) and a median prostate-specific antigen (PSA) level of 15 ng/ml (range 9.92–36.2). According to the D'Amico criteria, all ten patients had a high-risk cancer. Table 1 summarizes the characteristics of the examined patient cohort. The evaluation was done with approval of our local ethic committee (*S-321*). All patients signed a written informed consent form for the purpose of anonymized evaluation and publication of their data.

**Table 1:** Patient characteristics of the analyzed cohort

Patient #	Age	D'Amico Classifikation	GSC	PSA	PCa [SUVmax]	PCa[SUVmean]
1	71	high-risk	9	13.8	8.2	5.9
2	74	high-risk	8	16.2	25.4	19.3
3	65	high-risk	7b	35.7	20.9	15.7
4	72	high-risk	9	9.62	18.5	13.8
5	70	high-risk	6	31.8	7.8	5.9
6	64	high-risk	7b	93	22.1	16.1
7	61	high-risk	7b	36.17	33.4	23.1
8	73	high-risk	9	14.4	54	39
9	68	high-risk	8	13.3	14	10
10	70	high-risk	8	15.7	21.3	17.2

### PET/CT with ^68^Ga-PSMA11

PSMA-PET/CT was performed on a Biograph 6 PET/CT scanner (Siemens, Erlangen, Germany) 60 ± 10 min after intravenous injection of ^68^Ga-PSMA11 (median 179 MBq, range 121–240 MBq). ^68^Ga-PSMA11 was synthesized as previously described [10]. First, a CT scan (130 keV, 80 mAs; CareDose) without contrast medium was performed. Static emission scans, corrected for dead time, scatter and decay were acquired from the vertex to the proximal legs, requiring eight bed positions with 3 min per bed position. The images were iteratively reconstructed including CT-based attenuation correction with the OSEM algorithm using four iterations with eight subsets and Gaussian filtering to an in-plane spatial resolution of 5 mm at full-width at half-maximum. For calculation of the standardized uptake value (SUV), circular regions of interest were drawn around the area with focally increased uptake in transaxial slices and automatically adapted to a three-dimensional volume of interest with e.soft software (Siemens) at a 50 % isocontour.

### Multiparametric MRI

Magnetic resonance imaging was performed using T2w (TR: 5.68 ms, TE 84 ms, Matrix: 512 × 384), DWI (TR 2.806 ms, TE 75 ms, Matrix: 128 × 128, b-value 50, 400, 800 s/mm^2^), apparent diffusion coefficient (ADC)-map and T1w contrast enhanced MRI (FLASH 3D FS, TR 3.68 ms, TE 1.29 ms, Matrix 384 × 238) at 1.5 Tesla.

### Histopathological evaluation

The urologist/pathologist reported core biopsy with an eight-segment resolution. Reported as left/right lobe, basal/ apical level and central/ peripheral zone.

### Blind read and statistical analysis

**Fig. 1:**
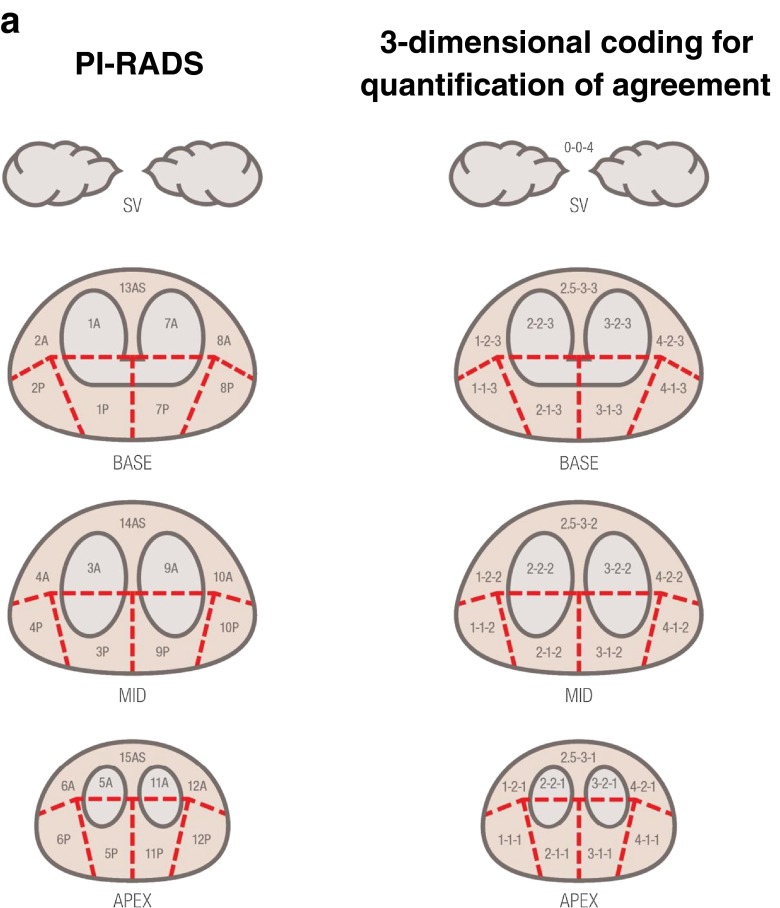

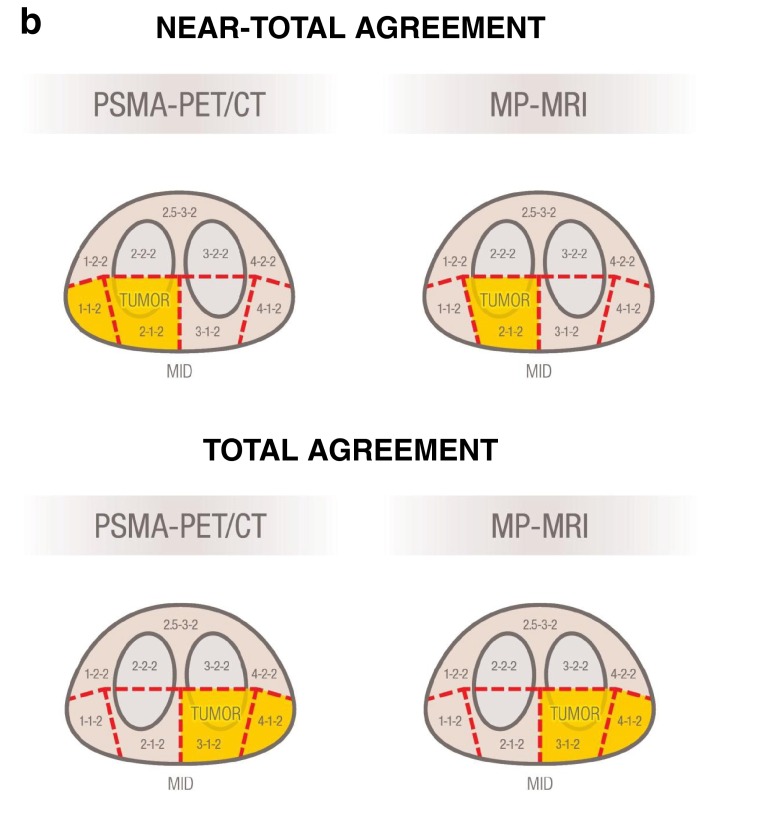
**a**: Blinded read evaluation according to PI-RADS and three-dimensional coding for quantification of agreement. The *left* figure presents the prostate regions as defined by PI-RADS, while the *right* figure visualizes our numerical coding for the three-dimensional position of these regions. **b**: The two figures present an example of an affected area in the prostate gland with near-total (*upper row*) and total (*low row*) agreement. In the *upper row*, all MRI-positive regions (section 2-1-2 only) were also found by PSMA-PET, while only half the PSMA-PET positive regions were identified identically in MRI. Thus, the total agreement score is 100/50 %. Regarding the section 1-1-2 missing in the MRI, an adjacent section (2-1-2) was identified by the MRI, so this is counted as a near-total agreement. Thus, the near-total agreement score is 100/100 %. In the *lower row*, all sections were identified identically by both methods; therefore, both agreement scores are 100/100 %

Two experienced readers (CK and HS) staged the tumor extension in the prostatic gland based on the PI-RADS scheme (Fig. 1a). For further statistical analysis, each sector of the PI-RADS scheme was represented in a three-dimensional coordinate system (x,y,z), with x representing the left/right orientation of the segment (from 1 to 4), y representing the position on the dorsoventral axis (from 1 to 3) and z representing the four different layers of apex, mid, base and semilunar vesicles (SV) (as 1–4). As there is only one central segment each in the ventral regions (13as/14as/15as), this segment has been assigned an x value of 2.5 to indicate its equal proximity to the adjacent *x* = 2 and *x* = 3 segments (Fig. 1b). Thus, for example, segment 9a is the third segment from the left, the second from the front, and on the second layer (mid), and is thus classified as (3-2-2), while 13as is classified as (2.5-3-3). As SV includes only a single section that is not adjacent to any other and infiltration of SV directly changes T-stage, we classified this segment as (0-0-4).

As neither of the two methods compared here is a definite gold standard, we consider two different analyses: First, we investigate what proportion of the sections identified by MP-MRI was also found in the PSMA-PET (MRI→PET), and second, what proportion of sections identified by PSMA-PET was also found by MP-MRI (PET→MRI). As an example, a result of (100/100 %) would indicate a perfect agreement, while a result of (100/50 %) would indicate that while the PSMA-PET confirmed all of the affected regions from the MP-MRI, it also identified twice the number of affected regions in total.

For both comparisons, we also performed a secondary analysis allowing partial agreement in the previous analysis. Partial agreement for an affected section was considered to be achieved if the second method identified either exactly the same section or a directly adjacent section (mathematically: Euclidian distance on the x,y,z coordinate system is one or less). Note that sections dislocated by one on two or more of the axes simultaneously were not considered to be adjacent (i.e., 3A is not adjacent to 9P).

## Results

All ten patients have been evaluated by the two-blinded readers based on MP-MRI and PSMA-PET/CT, respectively. The median SUVmax was 21.1 (range: 8.2–33.4) in the cancer affected area of the prostate gland according to the biopsy.

In comparison to the histological gold standard, both modalities found the main tumor mass in the location of the core biopsy containing the highest percentage of tumor involvement and Gleason score. As the “resolution” of tissue sampling in biopsy (eight segments—left/right, basal/apical, central/peripheral) is below the resolution of diagnostic imaging, histopathology could not serve as gold standard for tumor extension.

**Fig. 2:**
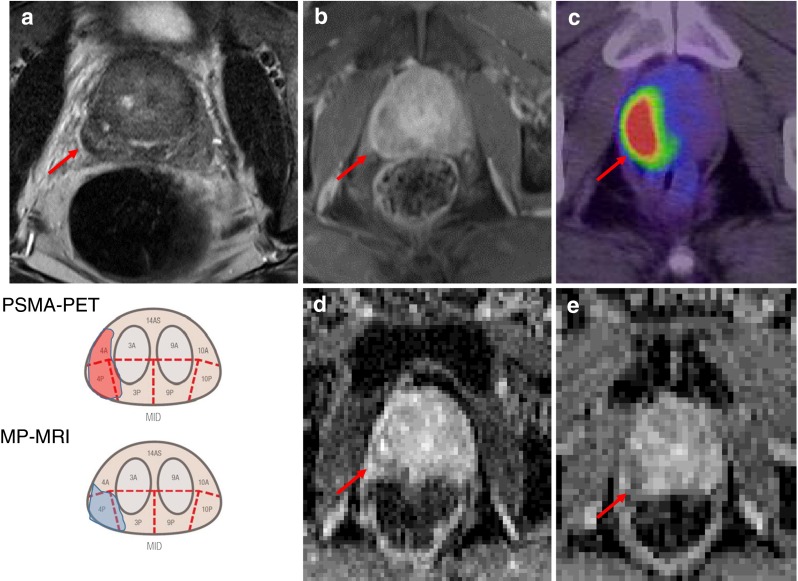
Example for a near-total agreement. Biopsy proven prostate cancer in the right, peripheral zone. In MP-MRI, a signal drop in T2w (**a**), fast wash-out in contrast enhanced T1w sequence (**b**), focal diffusion restrictions in DWI (**d**) and corresponding ADC-map (**e**) were interpreted as tumor infiltration of segment 4P. On a visual basis, the PSMA-PET (**c**) presents a very similar tumor extent, but was scored as tumor spread into the segments 4A and 4P

Despite the perfect concordance in eight-segment resolution, the tumor extent scored by the blinded readers using the PI-RADS schema presented some mismatch in 50 % of the cases (*n* = 10 patients); Fig. 2. The total agreement rates based on segment analysis (*n* = 270 segments) were MRI→PET 63.5 % (SD 31.1 %) and PET→MRI 80.2 % (SD 31.2 %), i.e., a bit more than three-fifths of the affected segments found in MP-MRI were also identically found in PSMA-PET, while four-fifths of the segments found in PSMA-PET were also found in MP-MRI.

**Fig. 3:**
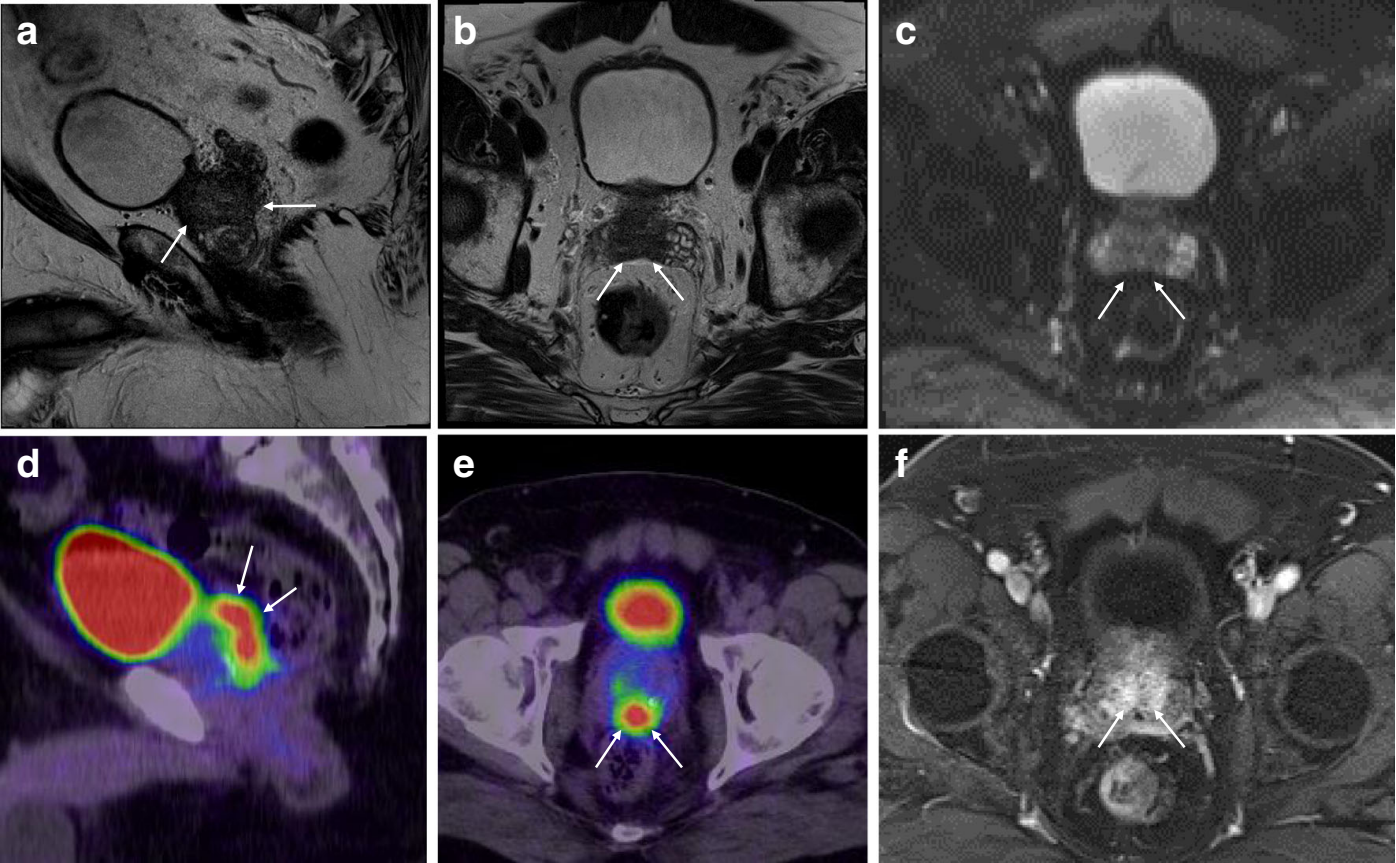
A 68-year-old patient presented a large extension of tumor mass in the base of the prostate gland with infiltration of the semilunar vesicles. In MP-MRI, this presents with a signal drop in T2w sequence (**a**, sagittal view; **b**, axial view), contrast enhancement in T1w sequence (**f**) and diffusion restriction, as demonstrated in the ADC map (**c**). PSMA-PET (***d*** *, sagital view;* ***e*** *, axial view*) presented with corresponding tumor spread

Near-total agreement rates were MRI→PET 89.4 % (SD 14.3 %) and PET→MRI 96.8 % (SD 7.1 %), i.e., in most cases for sections identified in one method, at least an adjacent section was identified by the other method. Only about 11 % of sections identified in MP-MRI could not be identified at all in PSMA-PET, and about 3 % of PSMA-PET results could not be confirmed at all in MP-MRI. Note that in five of the ten patients, a perfect agreement was achieved between both methods, including one patient with total agreement with regard to suspicious semilunar vesicle infiltration (Fig. 3).

## Discussion

We evaluated the potential of ^68^Ga-PSMA11-PET/CT for imaging primary PCa by intra-individual comparison to MP-MRI. As shown recently, PSMA-PET/CT has considerable impact on the radiotherapeutic management of prostate cancer patients [5]. Sensitive and specific imaging is a fundamental condition for definition of the target volume in radiotherapy. For example, radiation oncology aims to escalate dose to areas of high tumor cell burden and/or radiation resistance. Thus, targeting so called dominant intraprostatic lesions is currently under clinical investigation to enable individualized aggressive treatment based on personalized risk assessment [11].

Based on the eight-segment resolution of biopsy, MP-MRI and PSMA-PET/CT presented identical tumor allocations. However, these good results might have been supported by patient selection, which were all “high-risk” with a high probability for large T3/4 tumors. Nevertheless, according to the established 27-segment PI-RADS (V1) schema, total agreement was only found in 5/10 patients. However, the formally discrepant findings revealed to be near-total findings using the Euclidian distance to quantify translocation. Different anatomical landmarks delineable on CT and MRI might be one explanation, the higher in plane spatial resolution of MRI in comparison to PET, another. Nevertheless, measuring the exact tumor extension is a general challenge in PCa. PCa commonly presents with a mixture of different gradings simultaneously within one lesion as well as multi-locally. Blurred lesion borders appear due to a diffuse infiltrative growth. These challenges are present even microscopically, leading to recent revisions on the pathological classification criteria [12]. In analogy, amongst different MRI sequences, the measured lesion size can differ, e.g., hot-spots with strong diffusion restriction on DWI (Fig. 2d) are commonly interpreted to be tightly packed, cell-rich sub-areas within the tumor, but their extent can be significantly lower than the tumor size delineable in contrast enhanced T1w (Fig. 2b). In a similar manner, PSMA-PET might underestimate the total tumor extension. High PSMA expression is significantly correlated with higher Gleason score [13]. Thus, PET might highlight only poorly differentiated sub-volumes within the tumor, which might underestimate gross tumor diameter, but vice versa could be beneficial with regard to the described “dose painting” concepts in radiation therapy. However, this thesis has to be proven in the future. Another critical aspect that did not yet become apparent in our study is the possibility of PSMA negative tumor phenotypes, which have been reported with a probability of about 10 % in recurrent prostate cancers [3, 4]. Robust data for newly diagnosed PCa are still pending.

**Fig. 4:**
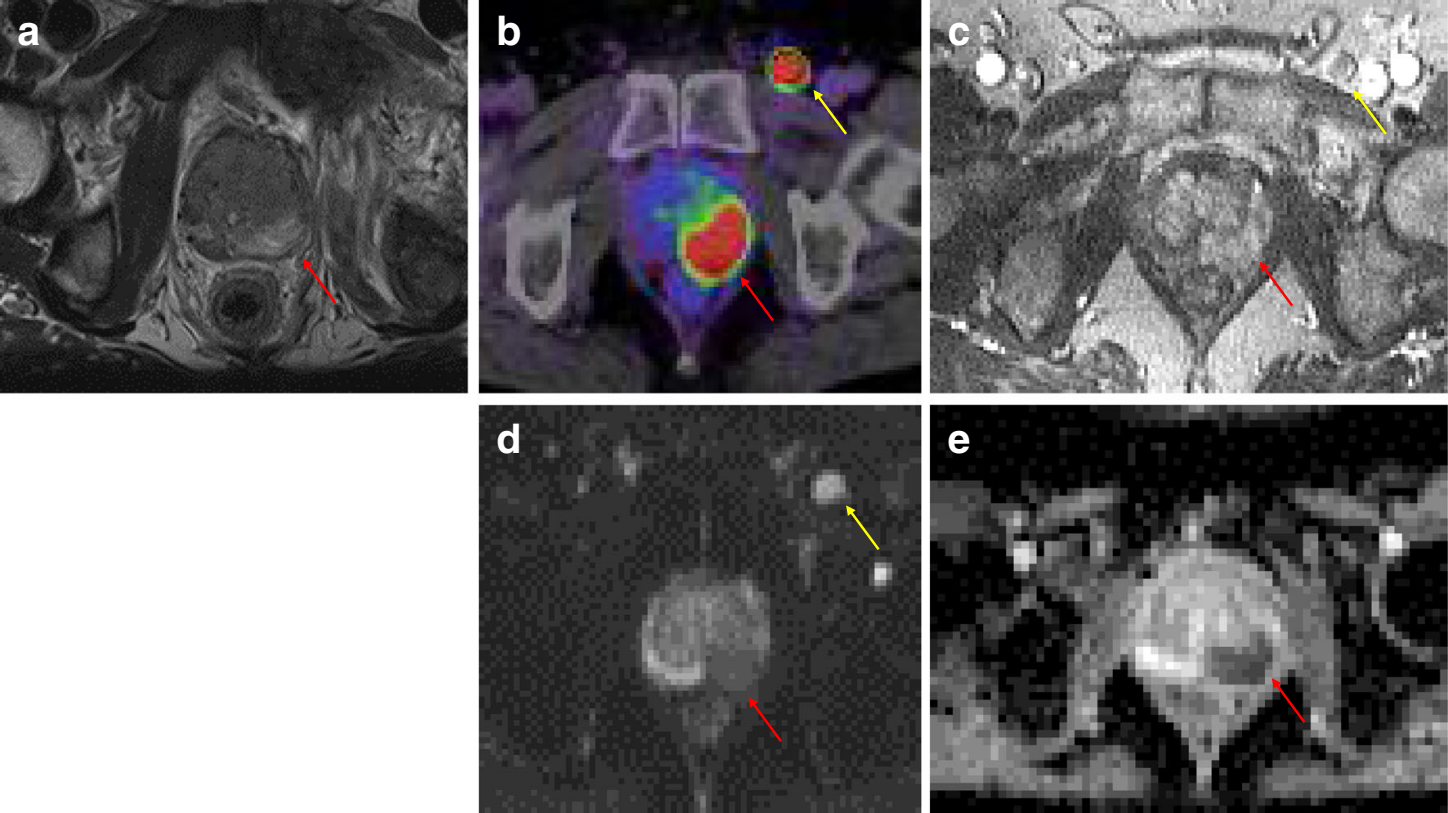
A 61-year-old patient with a biopsy-proven prostate cancer in the left peripheral zone. According to MP-MRI (**a**, T2w; **c**, CE-T1w; **d**, DWI, **e**, ADC), the tumor affected PI-RADS segments 9Pand 10P. In PSMA-PET (**b**, *red arrow*), a near-total agreement (tumor affecting PI-RADS segments 9P, 10P and 10A) was scored. PSMA-PET also diagnosed a loco-regional lymph node metastasis (**b**, *yellow arrow*) that was retrospectively also delineable in CE-T1w (**c**), DWI (**d**) and ADC (**e**)

The increasingly available of PET/MRI hybrid scanners might be suitable to combine the molecular information derived from PSMA-PET with the higher resolution and anatomical landmark definition derived from MRI. This novel generation of scanners may allow a “one stop” imaging for primary tumors (T-stage) by MP-MRI, while N- and M-staging can simultaneously be improved with PSMA-PET (Fig. 4).

In conclusion, PSMA-PET/CT and MP-MRI correlated well in tumor allocation in patients with a high pre-test probability for large tumors. Further research will be needed to evaluate the advantages of PSMA-PET/CT in challenging situations (e.g., prostatitis, benign prostate hyperplasia or after repeated negative biopsies) or if patients truly benefit from the handling of PSMA-positive findings as dominant intra-prostatic lesions during radiation therapy.
